# Randomised controlled trial of topical combination therapy chlorhexidine 0.2% and natamycin 5% versus topical natamycin 5% alone for fungal keratitis in East Africa: study protocol

**DOI:** 10.12688/wellcomeopenres.21390.1

**Published:** 2025-03-28

**Authors:** Jeremy John Hoffman, Simon Arunga, Einoti Matayan, Abel Ebong, Francis Orishaba, William Makupa, Muna Elisante, Reena Yadav, Sandip Das Sanyam, Tara Mtuy, David Macleod, Astrid Leck, Victor H Hu, Matthew J Burton

**Affiliations:** 1 London School of Hygiene & Tropical Medicine, London, UK; 2 Kilimanjaro Christian Medical Centre, Moshi, Tanzania; 3 National Institute for Health Research Biomedical Research Centre for Ophthalmology at Moorfields Eye Hospital NHS Foundation Trust and UCL Institute of Ophthalmology, London, UK; 4 Mbarara University of Science and Technology, Mbarara, Western Region, Uganda; 5 Sagarmatha Choudhary Eye Hospital, Lahan, Eastern Development Region, Nepal

**Keywords:** Clinical trial, fungal keratitis, chlorhexidine, natamycin, corneal ulcer, East Africa, microbial keratitis

## Abstract

**Introduction:**

Fungal corneal infection (fungal keratitis [FK]) poses significant treatment challenges. The efficacy of current topical antifungals is inconsistent and often limited, especially in low and middle-income countries where the majority of FK cases occur. Topical natamycin 5% is the current primary treatment in many countries, however, a substantial proportion of cases develop progressive disease, even with intensive treatment. Given the limitations of existing antifungal treatments, there is a need for alternative treatment strategies to address this condition.

Chlorhexidine, an antiseptic with both antibacterial and antifungal properties, has received attention as a potential therapeutic agent. While a recent randomized controlled trial (RCT) in Nepal demonstrated the superiority of natamycin over chlorhexidine, a pilot study in Uganda has indicated a possible role for adjunctive chlorhexidine 0.2% in FK treatment. The contrasting findings necessitate a comprehensive RCT to investigate the potential benefit of adding topical chlorhexidine 0.2% alongside natamycin 5% in the management of FK.

**Methods:**

We will test the hypothesis that topical natamycin 5% in combination with chlorhexidine 0.2% is superior to topical natamycin 5% alone in a two-arm, single-masked RCT (ISRCTN, ISRCTN87195453, registered 27/08/2020, https://www.isrctn.com/ISRCTN87195453). Participants are adults with FK presenting to tertiary ophthalmic hospitals in Tanzania and Uganda. Baseline assessment includes history, examination, photography, in vivo confocal microscopy and corneal scrapes for microbiology. Participants will be randomised to alternative topical antifungal treatments (topical chlorhexidine 0.2% and topical natamycin 5%; 1:1 ratio, 2-6 random block size). Patients will be reviewed at days 2, 7 (with re-culture), 14, 21, month 2, and month 3. The primary outcome is best spectacle corrected visual acuity (BSCVA) at three months. Primary analysis (intention-to-treat) will be by linear regression, with treatment arm and baseline BSCVA pre-specified covariates. Secondary outcomes include epithelial healing time, scar/infiltrate size, ulcer depth, hypopyon size, perforation and/or therapeutic penetrating keratoplasty, and positive re-culture rate.

## Plain language summary

This is the first randomised controlled clinical trial comparing natamycin 5% eye drops alone to natamycin 5% and chlorhexidine 0.2% combination eye drops for the treatment of fungal keratitis, an infection to the front of the eye that can lead to blindness if not treated appropriately. In this article we describe how we plan to conduct this clinical trial. The primary analysis will be a comparison by arm of best spectacle corrected visual acuity at 3 months, i.e. how well patients can see with glasses if needed at the end of the study period.

## Introduction

**Figure 1.**
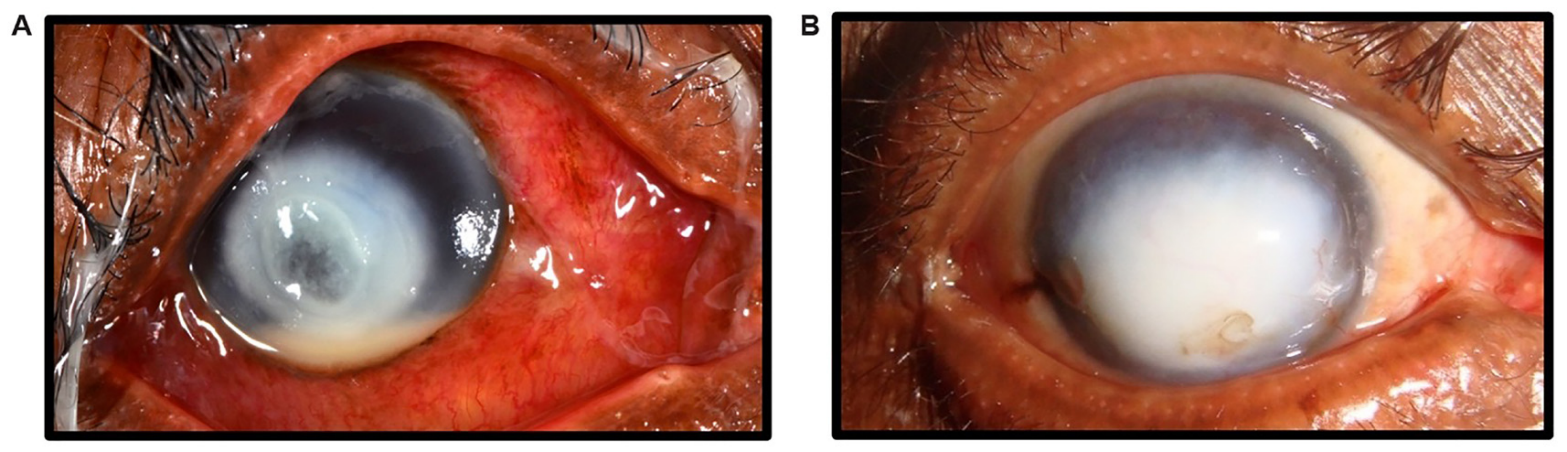
Fungal keratitis and corneal scarring. ( **A**) Active fungal keratitis with signs of acute inflammation and corneal ulceration. Photograph taken at presentation to SCEH. ( **B**) Corneal Scar, the blinding sequela of a resolved episode of fungal keratitis. Photograph taken at two months following presentation (same patient as ( **A**)). Consent for publication was granted from this patient.

**Figure 2.**
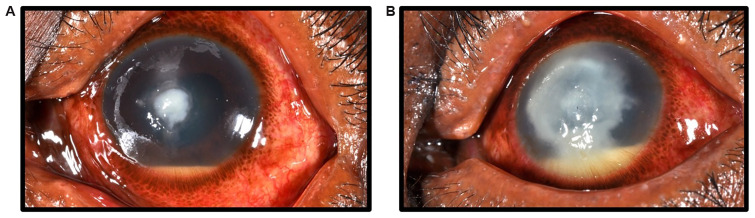
Progressive fungal keratitis. ( **A**) Early filamentous fungal keratitis; started immediately on intensive topical antifungal treatment (Natamycin 5%). ( **B**) The same case one week later, unresponsive to intense Natamycin 5% treatment, with progression of the infection. Consent for publication was granted from this patient.

Fungal keratitis (FK) presents a severe and potentially sight-threatening corneal infection, as depicted in Figure 1 ^1, 2^. This condition poses a significant burden, notably in tropical and subtropical regions, where factors such as climatic conditions (elevated temperatures and humidity) and frequent agricultural-related eye injuries contribute to its prevalence ^2, 3^. Accounting for 20–60% of diagnosed corneal infections in tropical regions, FK stands as a substantial subset of microbial keratitis (MK) ^4^. Regrettably, managing FK faces challenges compounded by limited treatment options, delays in timely interventions, and widespread misuse of inappropriate or alternative conventional medications, including topical corticosteroids and traditional eye remedies ^1, 5, 6^. Even when topical natamycin treatment, one of the standard options, is accessible, up to 30% of patients experience progression to corneal perforation or loss of vision, as demonstrated in Figure 2 ^1, 2, 7, 8^.

The primary approach to FK treatment typically involves frequent application of topical antifungal eye drops, reserving surgical intervention, such as therapeutic penetrating keratoplasty (TPK), for cases of corneal perforation or infection refractory to medical therapy. Antifungal medications fall into four main categories: imidazoles, triazoles, polyenes, and fluorinated pyrimidines, available in various forms, including topical, oral, intracameral, intrastromal, and intravenous administration. Notably, the treatment approach may differ for yeasts (Candida spp.) and filamentous fungi, with geographical variations in their relative proportions ^9, 10^.

Multiple clinical trials have compared FK treatment options. A meta-analysis favoured natamycin 5% over voriconazole ^11, 12^; hence, natamycin 5% is generally considered the first-line treatment for filamentous FK. Natamycin was included in the WHO Essential Medicines List in 2017 for this indication. Nevertheless, even with this treatment, infections can advance to perforation and vision loss in approximately 25% of cases, particularly in regions where antifungal eye drops are scarce or financially inaccessible ^1, 2, 7, 8^.

Chlorhexidine, renowned for its broad-spectrum antimicrobial properties, has been utilised in ophthalmology for over three decades, primarily as an eye-drop preservative and for sterilising contact lenses. Studies have explored its potential in treating FK, with promising outcomes from in vitro experiments and pilot randomised controlled trials comparing chlorhexidine to natamycin. Notably, chlorhexidine 0.2% exhibited dependable antifungal activity and showed a more favourable response compared to natamycin 2.5% within a 5-day timeframe ^13, 14^. Although a systematic review suggested a potential superiority of chlorhexidine over natamycin in curing FK ^11^, the limited size of earlier trials prompted our group to conduct a randomised controlled trial comparing natamycin 5% to chlorhexidine 0.2% for FK treatment in Nepal, aiming to provide more conclusive evidence on the efficacy of these treatments ^15^. This trial found that patients receiving natamycin 5% had significantly better best spectacle-corrected visual acuity (BSCVA) at 90 days compared to those receiving chlorhexidine 0.2% (estimated mean difference in vision - -0.30 logMAR; 95% confidence interval [CI], -0.42 to -0.18; P < 0.001), providing evidence that natamycin 5% is superior to chlorhexidine 0.2% ^16^. Patients treated with chlorhexidine healed 39% more slowly than those treated with natamycin (P < 0.001). However, there was no significant difference in the re-culture positivity rate at day-7 between arms (P=0.233).

It is worth noting that there is substantial global variation in the pattern of fungal species causing fungal keratitis ^2^. Specifically, Nepal and East Africa exhibit distinct fungal aetiology patterns, limiting the generalisability of the Nepal study to the African context. For instance, in Nepal, the majority of causative organisms were Curvularia spp. compared to Fusarium spp. in Uganda and Tanzania ^2, 16– 18^. Moreover, there might be differences in fungal susceptibility profiles and patients’ immunogenetic responses between the two regions.

While natamycin 5% showed promising results in our recent trial in Nepal, several key considerations arise. Firstly, natamycin is not routinely available in East Africa, and even when it is, it is financially out of reach for many patients. Secondly, the trial in Nepal excluded all patients who were receiving or had recently received topical treatment with antifungals. Over 100 patients were excluded from recruitment as they were already on topical antifungal treatment (primarily natamycin 5%) and were mostly attending the tertiary referral centre due to deteriorating condition despite ongoing treatment. These patients sought second-line agents to treat their infection, presenting an ongoing challenge. Thirdly, we conducted a case series in Uganda of 13 patients with fungal keratitis who were failing treatment with natamycin 5%. These patients received adjunctive chlorhexidine 0.2%. Among the 12 who completed three months of follow-up, 9/12 (75%) healed with corneal scarring. The vision was 6/18 or better in 5 of these patients (41.7%) ^19^. A tertiary referral eye hospital in the UK employs chlorhexidine 0.2% in patients with severe filamentous fungal keratitis in addition to natamycin 5% monotherapy ^20^.

Considering these observations, we are exploring whether combined therapy with natamycin 5% and chlorhexidine 0.2% yields better BSCVA at 3 months compared to natamycin 5% alone.

### Objective

The primary objective of this study is to determine if topical chlorhexidine 0.2% in combination with topical natamycin 5% is superior to topical natamycin 5% alone for treating fungal keratitis, in terms of vision at three months. The secondary objectives are: (1) to determine whether there is a difference between combination chlorhexidine 0.2% and natamycin 5% therapy and natamycin 5% alone for secondary clinical outcomes: infiltrate/scar size, time to re-epithelialisation, positive re-culture rates at one week; and (2) to investigate the effect of the alternative treatments on the Quality of Life of participants.

This trial is a response to the expressed need from both clinicians and patients for a readily available and affordable treatment strategy for fungal keratitis that can improve the likelihood of a good outcome. Our recent work in Uganda has shown promise for chlorhexidine 0.2% as an adjunctive therapy ^19^. If chlorhexidine 0.2% eye drops, which are very cheap and easy to prepare (by simple aqueous dilution), in combination with natamycin, are found to be superior to natamycin alone, then this offers a realistic, contextually relevant and sustainable solution for this aspect of the complex problem of fungal keratitis. Therefore, a full-scale trial investigating chlorhexidine 0.2% as adjunctive therapy is warranted to provide the evidence for its use.

## Methods

### Trial design

We will test the hypothesis that topical chlorhexidine 0.2% and topical natamycin 5% is superior to topical natamycin 5% alone in a single-masked randomised controlled trial in East Africa.

### Trial summary

**Figure 3.**
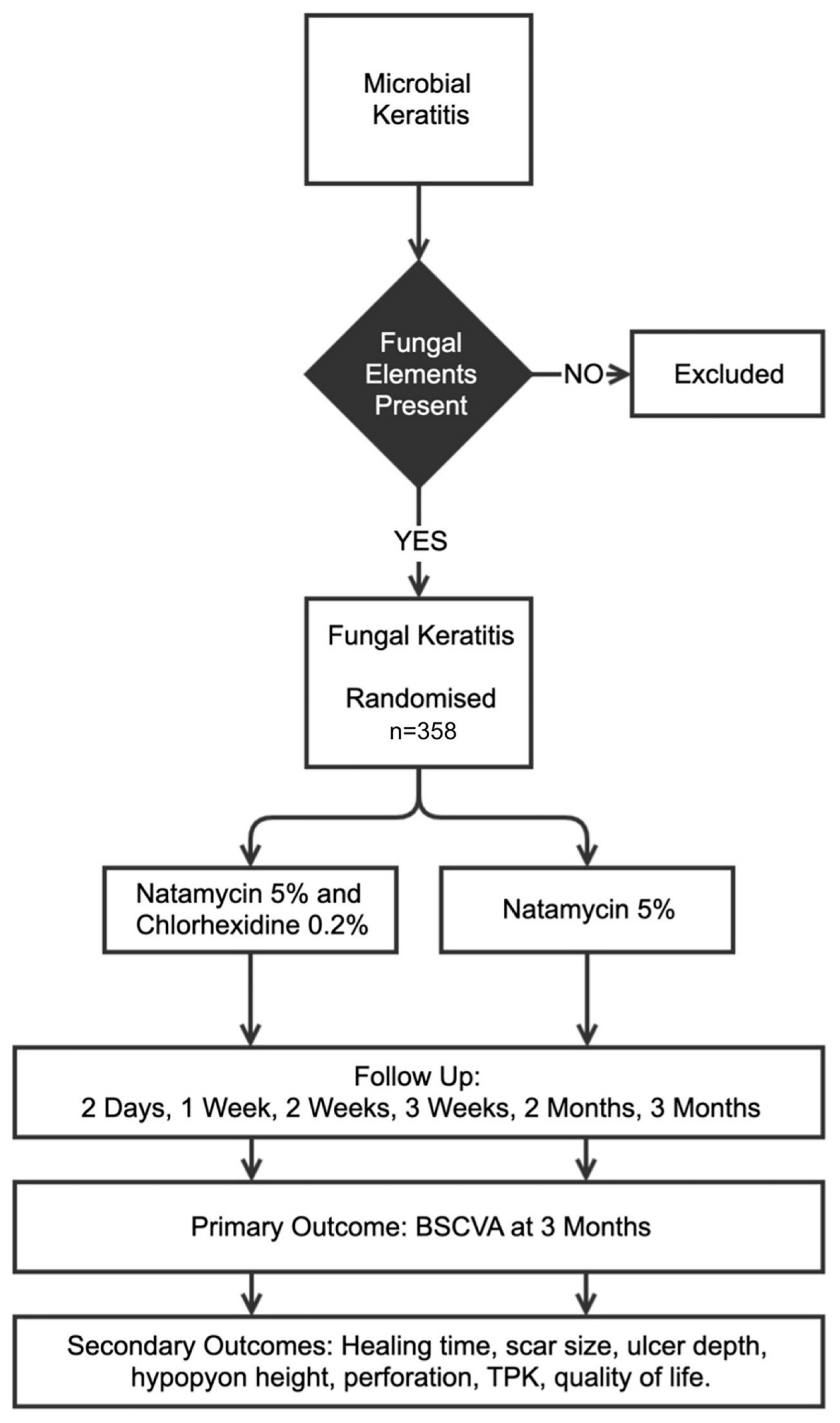
Overview of the clinical trial. Microbial keratitis is defined as presence of corneal epithelial ulceration (>1mm in diameter), corneal stromal infiltrate and signs of acute inflammation (e.g. conjunctival injection, anterior chamber inflammatory cells, hypopyon). Fungal elements to be detected by smear microscopy and/or confocal microscopy. Those eligible will be randomised 1:1 to natamycin + chlorhexidine or natamycin alone (n = 358). BSCVA: Best Spectacle Corrected Visual Acuity; TPK: Therapeutic Penetrating Keratoplasty.

This randomised controlled trial (RCT) adopts a two-stage recruitment process (see Figure 3), akin to the method previously outlined ^15, 16^. All patients presenting with acute microbial keratitis undergo assessment and enrolment into Stage 1 upon providing written, informed consent. This initial stage entails a comprehensive evaluation comprising history-taking, examination, and relevant investigations such as corneal scrapes for microbiological analysis and *in vivo* confocal microscopy. Should fungal hyphae be evident on smear or confocal microscopy, patients proceed to Stage 2. At this juncture, a trial eligibility assessment is completed, followed by Stage 2 written informed consent. We aim to enrol 358 patients into Stage 2. Eligible individuals with fungal keratitis (FK) are then randomly assigned in a 1:1 ratio to receive either chlorhexidine 0.2% in conjunction with natamycin 5% or natamycin 5% alone as topical ophthalmic treatments. Initially, treatment is administered hourly for the first week, followed by a two-hourly regimen for the subsequent two weeks. Subsequent treatment duration is adjusted based on clinical response. Personnel involved in the study remain blinded to the treatment allocation. Patients are typically admitted initially and undergo follow-up assessments on day-2, day-7 (including re-culture), day-14, day-21, month-2, and month-3. The primary outcome measure is the best spectacle-corrected visual acuity (BSCVA) at three months.

### Trial setting

This trial will be conducted in two centres in East Africa: Kilimanjaro Christian Medical Centre Hospital (KCMC), Moshi, Kilimanjaro Region of Tanzania and Department of Ophthalmology, Mbarara University of Science and Technology (MUST), Mbarara, Western Region, Uganda.

Both eye units serve large regional populations and frequently see patients with microbial keratitis, half of which is attributable to fungal infection ^1, 21^. The eye units at MUST and KCMC treat approximately 900 and 300 cases annually, respectively. It is anticipated that the study participants will present to the hospitals from multiple districts and regions within their respective countries. Individuals will be recruited from the outpatient clinics.

### Eligibility criteria

Prospective participants must fulfil all the inclusion criteria while not meeting any of the exclusion criteria outlined in Table 1. In essence, they should exhibit active fungal keratitis, defined as acute microbial keratitis characterised by corneal epithelial ulceration (>1mm in diameter), corneal stromal infiltrate, and signs of acute inflammation (e.g., conjunctival injection, anterior chamber inflammatory cells, hypopyon), alongside evidence of a filamentous fungal infection detected through smear microscopy and/or in vivo confocal microscopy (IVCM). IVCM has proven instrumental in identifying cases of fungal keratitis in our recent Nepal trial ^16, 18^, thereby contributing to robust evidence supporting its diagnostic utility in filamentary fungal keratitis. Previous studies have reported sensitivities ranging from 85.7% to 89.2% and specificities from 81.4% to 92.7%, respectively ^22, 23^. Given that some patients will be enrolled based on IVCM results, which may not reliably detect most bacteria, a subset of patients with microscopically confirmed fungal infection may subsequently manifest mixed infections upon study enrolment, as bacterial cultures may yield positive results a few days post-enrolment. As per our prior investigations, this scenario is anticipated to encompass approximately 10% of cases ^16^. These patients are included in the study but are excluded from the primary analysis of the primary outcome (as elaborated below). Secondary analyses will encompass mixed infections.

**Table 1.** Inclusion and exclusion criteria for enrolment in Stage 1 (microbial keratitis cases) and Stage 2 (the randomised controlled trial).

Stage 1:	
Inclusion Criteria (all must be met):	Exclusion Criteria (any of the following):
1. Acute MK characterised by: • Corneal epithelial ulceration >1mm diameter • Corneal stromal infiltrate • Acute inflammation: e.g. conjunctival injection, anterior chamber inflammatory cells, hypopyon. 2. Adults (18 years and older) 3. Able to provide informed consent	1. Patients aged less than 18 years 2. Patients unable or unwilling to provide informed consent 3. Patients who do not have acute microbial keratitis or where there is a more likely alternative diagnosis
Stage 2:	
Inclusion Criteria (all must be met):	Exclusion Criteria (any of the following):
1. Acute MK characterised by: • Corneal epithelial ulceration >1mm diameter • Corneal stromal infiltrate • Acute inflammation: e.g. conjunctival injection, anterior chamber inflammatory cells, hypopyon. 2. Filamentous fungal hyphae visualised on smear microscopy and/or *in vivo* confocal microscopy. 3. Agree to be randomised to either treatment arm and are able to give informed consent 4. Agree to be followed up at 2 days, 1 week, 2 weeks, 3 weeks, 2 months and 3 months 5. Adults (18 years and older)	1. Unwilling/unable to participate in trial and/or attend follow-up 2. Aged less than 18 years 3. Pregnancy: self-reported, or by urine hCG pregnancy test if uncertain. 4. Breast feeding: self-reported 5. No light perception in the affected eye 6. Fellow eye visual acuity <6/60 7. Acanthamoebic infection visualised by smear microscopy or IVCM 8. Clinical evidence of herpetic keratitis 9. Known allergy to study medication (including preservatives) 10. Previous keratoplasty in the affected eye 11. Bilateral corneal ulcers 12. Nationals of another country 13. Very severe ulcers warranting immediate evisceration or conjunctival flap 14. Endophthalmitis

### Consent procedures

In this trial, there are two distinct consent stages: Stage 1, open to all adult patients diagnosed with microbial keratitis, and Stage 2, exclusively for fungal keratitis patients meeting the eligibility criteria. This two-stage approach facilitates baseline data collection from all potential patients before confirming a diagnosis of fungal keratitis. Eligible patients will receive a participant information sheet in their local language (Swahili in Tanzania and Runyankore in Uganda), and its contents will be verbally communicated to them. They will have the opportunity to ask questions and seek clarification as needed. If a patient expresses willingness to participate, they will be requested to review and sign the study consent form, or provide a thumbprint if unable to sign. The consent process will be overseen by a nurse, whose confirmation signature will be affixed to the form. For patients unable to read the documents, a second witness unrelated to the study is required to corroborate the consent process. Consent forms are provided and available online (see Data Availability Statement).

### Baseline assessment

The comprehensive baseline assessment is delineated in Table 2. This encompasses clinical examination, corneal photography, in vivo confocal microscopy, and the procurement of microbiology samples. Additionally, participants will complete quality of life questionnaires, namely EQ-5D,24 WHO/PBD-VF20,25 and WHOQOL-BREF,26 with scoring details provided in Table 2.

**Table 2.** Baseline assessment. Assessment performed at baseline with details of how they are made.

Assessment	Details
Visual Acuity	Visual acuity, including Presenting, Pin-Hole, and Best Spectacle Corrected Visual Acuity (BSCVA), will be assessed using an ETDRS Tumbling-E logMAR 3m chart (Precision Vision, USA) mounted on an ESC 2000 ETDRS LED Cabinet (Precision Vision, USA). A trial-certified optometrist will perform these assessments separately for each eye.
Contrast Sensitivity	Contrast sensitivity will be evaluated using the Peek Contrast Sensitivity smartphone application, installed on an Android device (Sony Xperia Z3 Compact smartphone, Sony, Japan) ^24^.
Clinical Photographs	Photographs of both corneas will be taken using a Nikon D7500 camera equipped with an AF-S Micro Nikkor 105mm lens and SB-200 flash units (Nikon, Japan). A standardized protocol will be followed to ensure consistency in images across time points. Magnification will be standardized, enabling measurements of epithelial defects and stromal infiltrates.
Slit-lamp examination	A slit-lamp biomicroscope will be used to examine the anterior segment of both eyes, conducted by an ophthalmic clinician experienced in microbial keratitis management. Particular focus will be given to: 1. Eyelids: trichiasis, lagophthalmos, facial weakness, Bell’s reflex. 2. Suppuration. 3. Conjunctival inflammation. 4. Corneal sensation. 5. Corneal epithelial defects (including dimensions and ulcer depth). 6. Corneal inflammatory infiltrate characteristics: depth, size, profile, color, edge pattern, texture, satellites. 7. Anterior chamber inflammation: inflammatory cells, hypopyon, endothelial plaque. 8. Relative afferent pupillary defect.
*In vivo* confocal microscopy (IVCM)	The Heidelberg Retinal Tomograph 3 (HRT3) in vivo confocal microscope will be used for cellular-level corneal examination, allowing detection of fungal hyphae ^22, 23^. Each examination will use a sterile, single-use disposable cap on the objective lens, changed between patients. Volume scans capturing 400 x 400 μm images over an 80 μm depth range will be conducted. Scanning begins at the ulcer center and continues at the superior, inferior, nasal, and temporal edges, from the corneal epithelium to the deepest affected area. Images will be reviewed during the examination.
Ocular Sample Collection	At baseline, corneal ulcer samples will be collected as follows: 1. **Microscopy and Culture**: Corneal scrapes will be obtained after administering preservative-free proxymetacaine eye drops (Minims). Sterile needles will be used to transfer specimens to glass slides for immediate Gram stain, KOH, and Calcofluor white analysis. Samples will also be inoculated onto blood, chocolate, and Sabouraud agar and broths for culture. 2. **PCR Analysis**: Two sterile swabs will be gently swept over the corneal ulcer surface and stored in a 2ml tube at -80°C for PCR-based pathogen detection, fungal sequencing, and point-of-care fungal infection tests. If swabs yield insufficient material, an additional corneal scrape may be taken. PCR sample analysis will not be included in the RCT report.
HIV Testing	All individuals with microbial keratitis will be offered HIV counseling and testing. Positive cases not linked to HIV care services will be referred appropriately. Testing will use the HIV Tri-Dot rapid diagnostic test (Tanzania: J. Mitra & Co., Pvt. Ltd., India; Uganda: Abbott Diagnostics, Japan).
Random Blood Glucose	Participants will be screened for diabetes using a random blood glucose test (finger-prick sample). In Tanzania, the HumaLyzer Primus (HUMAN Gesellschaft für Biochemica und Diagnostica mbH, Germany) will be used, and in Uganda, Code Free blood glucose test strips (SD Biosensors, Inc., Korea). Results above 6.1 mmol/L will prompt referral for formal evaluation of impaired glucose tolerance or diabetes mellitus ^25^.
Quality of Life Questionnaires	Participants with confirmed fungal keratitis will undergo additional baseline assessments to evaluate quality of life: 1. **Vision-Related Quality of Life (VRQoL) ^26^:** The WHO/PBD-VF20 tool will be used to assess visual impairment's impact, including mental well-being, dependency, and social functioning. This 20-question instrument includes subscales on visual symptoms, general functioning, and psychosocial aspects. Responses use a five-point scale, from "very good" to "very bad," with a total score out of 100 (higher scores indicate better VRQoL). This has been used in several vision-related studies ^27, 28^. 2. **General Health-Related Quality of Life (HRQoL):** The EQ-5D questionnaire and EQ-Visual Analogue Scale will assess general health ^29^. Additionally, the WHOQOL-BREF tool, developed for low- and middle-income countries ^30^, will measure four health domains: Physical Health, Psychological Health, Social Relationships, and Environment. It consists of 26 questions scored on a positive scale from 1 (low satisfaction) to 5 (high satisfaction). Domain scores are calculated as the mean of item scores and scaled for comparability with WHOQOL-100. Two separate items address overall quality of life and health perception. Higher scores indicate better quality of life.

### Randomisation and masking

***Sequence generation:***

- A computer-generated randomisation list will be created by an independent statistician at LSHTM. This statistician will maintain the sequence but will not be masked and will not participate in any other aspect of the study.
- The allocation ratio will be 1:1 for chlorhexidine + natamycin to natamycin, with blocked randomisation to ensure reasonable balance across different locations over time. Block sizes will randomly vary between 2, 4, and 6.
- Separate randomisation sequences will be generated for each of the two recruitment centres (Uganda and Tanzania).

***Allocation concealment and implementation:***

- Randomisation sequences will be concealed in sequentially numbered, opaque envelopes.
- An independent administrator, experienced in trial procedures, will prepare the envelopes and will not be involved in other trial aspects.
- A research nurse in each centre will open the envelopes sequentially and allocate treatment to participants.
- The randomisation administrator will be a nurse or pharmacist with appropriate training.
- Investigational products will be stored in a dedicated, locked drug cabinet in the trial coordination office, managed only by the randomisation administrator.
- Storage conditions specified by the manufacturer will be adhered to.
- The randomisation administrator will handle storage, transportation, and dispensing of drugs.
- Stock reconciliation will be conducted at the end of each recruitment day.

***Masking:***

- Due to differing appearances of the treatments, participants cannot be masked.
- Clinicians assessing patients will be masked to allocation.
- The primary analysis statistician will be masked to allocation until the analysis code is pre-tested.
- Optometrists assessing visual acuity at three months will be masked to allocation.
- Independent masked grading of photographs will confirm outcome measures and detect any potential bias from clinical examiners.

***Unmasking:***

- Unmasking will only occur if necessary for participant safety.
- Staff unmasked for data and safety monitoring committee analyses will not participate in other study aspects.
- A list of unmasked staff will be maintained and approved by the Chief Investigator.
- Unmasked staff must acknowledge their confidentiality responsibilities in writing.
- Processes for providing access to unmasked treatment codes and reports will be documented.

### Intervention and treatment

Patients diagnosed with fungal keratitis typically receive admission for close observation and supervised treatment until improvement is evident, and outpatient management is deemed safe by the supervising clinician.

### Trial treatment arms

A) **Combination Therapy:** Chlorhexidine 0.2% w/v eye drops and natamycin 5% w/v eye drops are administered to the infected eye (one drop per application), with chlorhexidine applied first followed by natamycin, five minutes apart. Initially, treatment is hourly for the first week, reduced to every two hours for two weeks if improvement is observed. Subsequent treatment duration and frequency are adjusted based on clinical response. The chlorhexidine 0.2% w/v eye drops used in these studies will be produced by Mandeville Medicines, UK. The natamycin 5% used will be from the same source as described below.

B) **Natamycin Monotherapy:** Natamycin 5% w/v eye drops are applied to the infected eye (one drop per application) hourly for the first week, then reduced to every two hours for two weeks if signs of improvement are noted. Subsequent treatment adjustments are made according to clinical response. Topical natamycin 5% will be sourced from the manufacturer Sun Pharmaceuticals, India, and supplied by GNH India.

### Dosing schedule

Both treatment arms follow the same dosing regimen. Hourly administration is maintained for 48 hours, followed by hourly administration while awake for five days, then two-hourly administration while awake for two additional weeks. Treatment discontinuation is considered if the ulcer heals, and adjustments are made if the ulcer resolves partially.

### Additional topical treatments

1. Fluorescein sodium ophthalmic strips: Used to highlight corneal epithelial defects (Tanzania: Contacare Ophthalmics and Diagnostics, India; Uganda: locally produced) ^31^.

2. Anaesthetic eye drops: Administered before procedures such as microbiology sampling. Proxymetacaine 0.5% eye drop Minims (Bausch and Lomb, UK).

3. Antibiotic eye drops: Used if bacterial/fungal mixed keratitis is suspected or for prophylaxis against secondary bacterial keratitis: Moxifloxacin 0.5% eye drops (Tanzania: Centaur Pharmaceuticals, India; Uganda: Abacus Parenteral Pharmaceuticals, Uganda)

4. Mydriatic eye drops: For pupil dilation to alleviate discomfort. Tanzania: Atropine 1% eye drops (Aurolab, India), twice daily; Uganda: Cyclopentolate 2% eye drops (Abacus Parenteral Pharmaceuticals, Uganda), three times a day.

5. Ocular hypotensive eye drops: Prescribed if intraocular pressure exceeds 25 mmHg. Usual first line treatment is timolol 0.5% eye drops (Tanzania: Allergan, India; Uganda: Abacus Parenteral Pharmaceuticals, Uganda).

### Ancillary treatment for refractory cases

Patients unresponsive to trial medication for seven days or more may receive additional treatments such as topical amphotericin B, oral ketoconazole, or intracameral amphotericin B, depending on ulcer depth and progression.

### Non-pharmacological treatment

Surgical interventions, performed by supervising consultant ophthalmologists, may include bandage contact lens insertion, tissue glue and patch application, or conjunctival flaps to manage small perforations or non-healing ulcers. Corneal transplant is not available in Uganda or Tanzania.

### Primary outcome measure

***Best Spectacle Corrected Visual Acuity (BSCVA) at Three Months:*** Measured in logMAR units by a trial-certified optometrist, independent and masked to allocation. This outcome is selected for its functional significance and comparability with prior trials, including our recent Nepal trial ^7, 16^. Three months is chosen as it aligns with clinical experience indicating typical healing time for corneal ulcers. BSCVA will be assessed using an LED-backlit, Tumbling-E LogMAR chart under controlled conditions. Peek Acuity, a validated smartphone application, will be used if hospital visits are not feasible ^32^. LogMAR values for patients with CF vision or less will be applied, as previously described ^33, 34^.

### Secondary outcome measures

- **Clinical Signs of Healing:** Including reduction of epithelial defect.
- **Microbiological Culture Rates:** Assessing for positive or negative cultures.
- **Other Clinical Outcome Measures:** Such as scar size or perforation rate.

### Outcome assessments

Participants will undergo reassessment at various intervals following enrolment, as outlined in Table 3. Examinations will be conducted similarly to the baseline assessment. Adherence to treatment and symptoms, including side effects, will be queried and recorded at each follow-up. Trial medication adherence will be monitored by weighing eye drop bottles at specified follow-ups, with resupply provided as necessary. Visual acuity will be measured at each visit, with BSCVA assessed at the three-month follow-up (primary outcome measure). Quality of life questionnaires will be repeated at the final three-month follow-up. Slit-lamp examination and corneal photography will be performed at each visit. In vivo confocal microscopy will be repeated at specified intervals to assess fungal hyphae resolution. If the ulcer has not healed at the one-week follow-up, re-scraping for repeat culture will be conducted. Appointment cards will be provided for follow-ups, and transport costs will be covered for outpatient participants.

**Table 3.** Baseline and follow-up assessment components.

Assessment Item	Baseline	Day 2	Day 7	Day 14	Day 21	Day 60	Day 90
History / Baseline questionnaire	X						
Check treatment adherence		X	X	X	X	X	X
Check for side effects		X	X	X	X	X	X
Visual Acuity – Presenting	X	X	X	X	X	X	X
Visual Acuity – BSCVA	X						X
Contrast Sensitivity	X						X
Slit-lamp Examination	X	X	X	X	X	X	X
Cornea Photography	X	X	X	X	X	X	X
*In vivo* confocal microscopy	X		X	X	X		
Cornea samples (Microbiology/PCR)	X		X				
Quality of Life Tools	X						X

### Treatment review

At each follow-up appointment, participants will be assessed by an ophthalmic clinician with expertise in fungal keratitis management. Clinical responses to antifungal treatments are generally slower compared to bacterial infections, often necessitating prolonged topical therapy for 4–6 weeks. As a result, adjustments to therapy are typically deferred for at least one week. Additional interventions may be required, including corneal glue application for perforations, conjunctival flap procedures, or therapeutic penetrating keratoplasty (corneal transplantation). 

### Stopping rules

If the study eye experiences a significant adverse event believed to be linked to the antifungal study medication, its use may be discontinued. The patient will then receive treatment as determined by the supervising ophthalmologist, without unblinding the randomisation code. Even if the study medication is stopped, the patient will continue scheduled follow-up visits. 

### Loss to follow-up

Low rates of loss to follow-up are anticipated based on prior clinical experience. Patients who miss follow-up visits will be contacted by phone, and reasons for non-attendance will be documented. Patients will be encouraged to attend for continued treatment and monitoring. If they are unable to visit the hospital due to illness or other barriers, home visits by the study team will be arranged. Reasons for loss to follow-up will be recorded and reported accordingly. 

### Data collection, management, confidentiality, and access to data

Data will be recorded on paper-based Clinical Record Forms (CRFs) securely stored at study sites. Scanned electronic copies will be saved daily on an encrypted drive, with backups made both on-site and off-site. Double data entry will be conducted into two separate MS Access databases, with data cleaning performed using EpiData version 3.1 (available for free at: https://www.epidata.dk). Local study coordinators will supervise data collection and entry daily, and progress will be reviewed weekly by the study coordinator and the chief investigator at LSHTM. Data confidentiality will be maintained by restricting database access and securing paper documents in locked cabinets accessible only to authorised personnel. The database will require password authentication, with individual passwords assigned to data entry staff. Anonymised datasets will be used for further analysis. 

### Data and Safety Monitoring Board

The Data and Safety Monitoring Board (DSMB) for the trial comprises independent experts in bioethics, biostatistics, epidemiology, and ophthalmology, appointed by the Trial Steering Committee and approved by regulatory authorities. The DSMB convenes before the trial and periodically thereafter, with additional teleconferences as required. The DSMB oversees the study protocol, modifications, severe or unexpected events, and the interim analysis results, determining whether the trial should continue as planned or with adjustments. Ethics committee approvals from Tanzania, Uganda, and LSHTM are prerequisites, and all changes undergo DSMB review. 

### Monitoring for harm

Patients will be monitored for adverse events or reactions during each visit, using standardised definitions outlined in Appendix 2. Reporting protocols for adverse events, unexpected adverse reactions, and serious reactions will also follow Appendix 2. 

### Biological specimens

Procedures for processing and analyzing biological specimens are detailed in Appendix 3. 

### Sample size considerations

The study is designed to test the hypothesis that natamycin combined with chlorhexidine is superior to natamycin alone in improving the primary outcome (BSCVA at 3 months). Assuming a BSCVA of 0.5 logMAR in the natamycin-only group, a standard deviation of 0.62 in both groups, and an adjusted alpha of 0.0492 (to accommodate interim analysis using the O’Brien-Fleming method), a sample size of 304 ensures 80% power to detect a 0.2 logMAR improvement. Allowing for a 15% dropout rate, the total target enrollment is 358 participants across both sites. 

### Analysis plan

An intention-to-treat (ITT) approach will be used, analyzing all data based on the assigned randomisation group, regardless of adherence to the treatment protocol. Analyses will follow CONSORT guidelines for non-inferiority trials ^35^, and a flowchart will summarize recruitment, randomisation, and follow-up by treatment arm. Baseline characteristics will also be summarized. Details of the SPIRIT checklist are provided as Extended Data and available online (see Data Availability Statement). 

***Primary outcome analysis – unadjusted***

The primary outcome (BSCVA at 3 months) will be analyzed using linear regression, including baseline BSCVA and treatment arm as covariates. Mixed fungal and bacterial infections identified at baseline will be excluded from the primary analysis. We will use our alpha of 0.0492 to test the null hypotheses at 0.0492 significance. Significance will be assessed using a two-tailed test at 0.0492 level for assessing superiority.

***Primary outcome analysis – adjusted***

If baseline covariate imbalances occur between treatment arms, adjusted analyses will be conducted to ensure treatment effects are not confounded by these differences. This is particularly important if natamycin + chlorhexidine has a better outcome than natamycin, as the adjusted treatment effects may account for this observed imbalance whilst the unadjusted analyses may not. Sensitivity analyses will allow us to show that any observed positive treatment effect is not solely explained by imbalances at baseline in any of the covariates.

***Secondary analyses of the primary outcome***

- Per-Protocol Analysis: Re-analysis will be conducted excluding participants with significant deviations, poor adherence (<50%), or mixed/non-fungal infections. 

- Mixed Infections: Secondary analyses will include mixed infections using the same methodology as the primary analysis. 

- Sensitivity Analyses: Missing data will be addressed through multiple imputation or non-random missing data models if necessary. Additional sensitivity analyses will assess outcomes in subgroups such as those with severe vision loss, corneal perforation, or therapeutic keratoplasty. 

***Analysis of other determinants for success***

Logistic regression models will identify factors associated with poor outcomes (BSCVA >1.0 logMAR), adjusting for trial arm. Multivariate models will refine predictors based on likelihood ratio tests. 

***Secondary outcome analysis***

Secondary outcomes listed in Table 4 will be analyzed using regression models appropriate for the data type, adjusting for any baseline imbalances. 

**Table 4.** Secondary Outcome Measures that will be investigated as part of the trial, together with analysis details.

Secondary outcome measure	Details
Three-week BSCVA	We will analyze the secondary outcome of three-week Best Spectacle-Corrected Visual Acuity (BSCVA) in logMAR using the same methodology applied to the primary analysis of the primary outcome. The three-week BSCVA assessment will include values recorded between 18 days and 5 weeks, with the closest value to 3 weeks being selected.
Presenting VA by Peek	The presenting visual acuity (VA) assessed using Peek Acuity, with and without pinhole, will also be analyzed as a secondary outcome at three months. This measure will be particularly relevant if reliable BSCVA readings cannot be obtained at three months (e.g., if patients do not attend the clinic and at-home visual acuity testing is required). This analysis will follow the same approach as the primary analysis of the primary outcome. A sensitivity analysis, incorporating data from participants lost to follow-up, will use the most recent recorded observation of this variable.
Scar/infiltrate size at 1 week, 3 weeks and 3 months by slit lamp examination.	For scar or infiltrate dimensions, the geometric mean of the two principal axes (in mm) will serve as the outcome variable at one week, three weeks, and three months. The scar size, observed via slit lamp, will be compared between treatment groups at each time point using linear regression. This will include treatment arm and baseline infiltrate/scar size as predefined covariates, controlling for baseline differences.
Time to full epithelial healing (slit lamp examination by ophthalmic clinician)	The time to re-epithelialization will be calculated as the midpoint between the last review showing an epithelial defect (ED) and the subsequent review where no ED is observed. Fluorescein staining covering an area smaller than 0.5 mm will be considered indicative of a resolved ED, given the challenge of distinguishing smaller defects from pooling in healed areas. Time-to-healing analysis will utilize Cox proportional hazards regression, with treatment group as the primary predictor and baseline ED size (geometric mean in mm) included as a covariate. Survival curves for treatment groups will be plotted using Kaplan-Meier analysis up to the three-month endpoint. The proportional hazards assumption will be tested by stratifying baseline ED size into quartiles. If this assumption is not met, stratified results will be reported. Treatment failure (defined as an ED larger than 0.5 mm at the three-month review) will be compared between groups using Fisher’s exact test.
Rate of healing	The rate of ulcer size reduction will be evaluated by measuring changes in ED size over time intervals (1 week to 3 weeks, and 3 weeks to 3 months) and dividing by the number of days, producing a rate (mm/day). This will be analyzed using Cox regression.
Microbiological cure	For patients with persistent corneal ulcers (defined by ED presence) at day 7, a repeat corneal scrape and microbiological tests will be conducted. Microbiological cure at day 7 will be defined as the absence of growth on culture. Cure rates will be compared between treatment arms using logistic regression, adjusting for organism type (e.g., *Aspergillus spp.*, *Fusarium spp.*, or other).
Ulcer depth at 1 week and 3 weeks (slit lamp examination by ophthalmic clinician).	Depth of corneal ulcers (expressed as a percentage of healthy cornea thickness) will be compared between treatment groups at one week and three weeks. This analysis will adjust for baseline ulcer depth using linear regression.
Hypopyon height at 1 and 3 weeks, (slit lamp examination by ophthalmic clinician)	Hypopyon height (in mm) at one and three weeks will be compared between groups, adjusting for baseline height using linear regression.
Perforation and/or TPK and/or conjunctival advancement by three months (slit lamp examination by ophthalmic clinician)	The number of patients requiring perforation repair via Therapeutic Penetrating Keratoplasty (TPK), conjunctival advancement, or experiencing perforation itself by three months will be reported using confidence intervals and descriptive statistics. Since the study is not powered to detect significant differences in perforation or TPK rates, exploratory logistic regression analyses will estimate odds ratios for these outcomes between groups.
Loss of Eye	The proportion of patients requiring surgical removal of the eye (evisceration or enucleation) during follow-up will be described with confidence intervals. Logistic regression will explore treatment arm differences and associated risk factors.
Ocular adverse effects, slit lamp examination by ophthalmic clinician.	TThe occurrence of adverse events will be assessed by Fisher’s exact test. Poisson regression will compare adverse event rates, accounting for multiple events per participant.
Quality of life assessed using: EQ-5D, WHO/PBD-VF20, WHOQOL-BREF	QoL outcomes will be assessed using tools tailored to specific interests ^38^. Disease-related QoL will be evaluated with the WHO/PBD-VF20 (20-item Vision Function Questionnaire), which assesses the impact of visual impairment on mental well-being, dependency, and social functioning. For general health-related QoL, tools include the EQ-5D questionnaire, EQ-Visual Analogue Scale, and the WHOQOL-BREF. The EQ-5D provides standardized health outcome measures, while the WHOQOL-BREF evaluates four domains: Physical Health, Psychological Health, Social Relationships, and Environment. Scoring details for these tools are provided in Table 2. Comparisons of QoL scores between treatment groups will estimate the effects of chlorhexidine and natamycin. Adjustments will be made for variables like age, sex, socio-economic status, and recent health problems. Logistic, linear, and ordinal logistic regression methods will be employed depending on whether the outcome variables are binary, continuous, or ordered categorical. Mean scores and mean differences in QoL subscales and domains will be compared using t-tests and linear regression.
Cost effectiveness analysis, using EQ-5D data from 3 months and direct cost data	Direct costs incurred by patients will be collected at the three-month follow-up, with economic costs estimated from EQ-5D data collected at baseline and follow-up. Mean direct costs will be compared between treatment arms using t-tests. Changes in EQ-5D scores from baseline to follow-up will also be analyzed similarly.
Drug adherence	The rate of adherence to treatment protocols will be evaluated using descriptive statistics, comparing compliance between the two treatment arms.

***Interim analysis***

An independent statistician will conduct an interim analysis after 1/3 of participants complete follow-up. 

### Patient and Public Involvement

Pre-study discussions in Tanzania and Uganda highlighted delays in care-seeking for fungal keratitis due to treatment costs, availability, and perceived inefficacy ^36, 37^. Community health workers emphasized the need for further training and governmental support to enhance care delivery. 

## Ethics and consent

This work will adhere to the tenets of the Declaration of Helsinki. Ethics committee and regulatory review and approval have been obtained from the Kilimanjaro Christian Medical University College Moshi, Tanzania (approval number 2431) (Date of approval: 14/08/2019); Mbarara University of Science and Technology, Mbarara, Uganda (approval number MUREC 1/7) (Date of approval:05/09/2019); National Institute for Medical Research, Dar es Salaam, Tanzania (approval number NIMR/HQ/R.8a/Vol. IX/3091) (Date of approval: 14/05/2019); Uganda National Council for Science and Technology, Kampala, Uganda (approval number HS 2514)(date of approval: 05/08/2019); the National Drug Authority, Kampala, Uganda (approval number CTC - 0138/2020); the Tanzania Medicines and Medical Devices Authority, Dodoma, Tanzania (approval number TMDA0019/CTR/0024/02); and the London School of Hygiene and Tropical Medicine Ethics Committee, UK (approval number 14908) (Date of approval: 04/04/2018). The study is registered with ISRCTN (87195453, Table 5). The trial Sponsor is the London School of Hygiene and Tropical Medicine. Any protocol modifications will be submitted for review. Patients requiring continued care post-study will receive treatment at study centers. Results will be presented at scientific meetings and published in peer-reviewed journals.

**Table 5.** Registration Data and Protocol Summary.

Data category	Information
Primary registry and trial identifying number	ISRCTN Registry; ISRCTN87195453
Date of registration in primary registry	27 August 2020
Secondary identifying numbers	
Source(s) of monetary or material support	Wellcome Trust
Primary sponsor	London School of Hygiene and Tropical Medicine (LSHTM)
Secondary sponsor(s)	
Contact for queries	Jeremy Hoffman PhD FRCOphth ( Jeremy.hoffman@lshtm.ac.uk)
Title	A comparison of two treatment regimes for the treatment of fungal eye infections in East Africa
Countries of recruitment	Tanzania and Uganda
Health condition(s) or problem(s) studied	Fungal keratitis
Intervention(s)	Participants will be randomised to either topical natamycin 5% plus chlorhexidine 0.2% or topical natamycin 5% alone
Key eligibility criteria	1. Acute MK characterised by: • Corneal epithelial ulceration >1mm diameter • Corneal stromal infiltrate • Acute inflammation: e.g. conjunctival injection, anterior chamber inflammatory cells, hypopyon. 2. Filamentous fungal hyphae visualised on smear microscopy and/or *in vivo* confocal microscopy. 3. Agree to be randomised to either treatment arm and able to give informed consent 4. Agree to be followed up at 2 days, 1 week, 2 weeks, 3 weeks, 2 months and 3 months 5. Adults (18 years and older)
Study type	Randomised controlled trial
Date of first enrolment	12 May 2021
Target sample size	358
Recruitment status	Recruiting
Primary outcome(s)	Best Spectacle Corrected Visual Acuity (BSCVA) at 3 months by a trial certified optometrist
Key secondary outcomes	1. Time to full epithelial healing (slit lamp examination by ophthalmic clinician). 2. Pin-hole visual acuity in logMAR at 3 months, trial-certified optometrist 3. Scar/infiltrate size at 1 week, 3 weeks and 3 months (slit lamp examination by ophthalmic clinician). 4. Ulcer depth at 1 week and 3 weeks (slit lamp examination by ophthalmic clinician). 5. Hypopyon height at 1 and 3 weeks, (slit lamp examination by ophthalmic clinician). 6. Perforation and/or TPK by three months (slit lamp examination by ophthalmic clinician). 7. Positive culture rate at 1 week 8. Ocular adverse effects at each follow up visit (Day 2, Day 7, Day 14, 3 weeks, 2 months, 3 months), slit lamp examination by ophthalmic clinician. 9. Quality of life (QoL) assessed using: EQ-5D, WHO/PBD-VF20, WHOQOL-BREF (comparison between baseline and QoL measures at 3 months) 10. Cost-effectiveness analysis, using EQ-5D data from 3 months and direct cost data. 11. Drug adherence at each follow up visit (Day 2, Day 7, Day 14, 3 weeks, 2 months, 3 months) whilst the patient is using study medications.

Any patients who may be eligible to participate will be given a participant information sheet and its contents will be read out to them by a study team member. They will be asked if they would be willing to participate, which involves being randomised to alternative treatment arms and to be followed-up for three months. The patient will then have the opportunity to discuss any questions that they might have. If the patient would like to participate, they will be asked to read and sign or place a thumb print on the study consent form. The consent will be witnessed by the clinic nurse by a signature on the form. For patients who are unable to read the documentation a second witness who is unrelated to the study will be required.

## Data Availability

No data associated with this article LSHTM Data Compass: Appendix files for "Randomised controlled trial of topical combination therapy chlorhexidine 0.2% and natamycin 5% versus topical natamycin 5% alone for fungal keratitis in East Africa". https://doi.org/10.17037/DATA.00004598 ^39^. Appendix1-PIS_consent-East_Africa_2 Appendix2-Monitoring_for_harm Appendix3-Biological_specimens Appendix4-Spirit_checklist Data are available under the terms of the Creative Commons Zero "No rights reserved" data waiver (CC0 1.0 Public domain dedication).

## Data statement

The datasets generated and/or analyzed during this study will be accessible upon request from Prof. Matthew Burton ( matthew.burton@lshtm.ac.uk). The data provided will be fully anonymized, with all patient-identifiable information removed. Access to the full dataset will be granted only after the formal reporting of study findings in a peer-reviewed scientific journal.

Datasets will be available exclusively to bona fide scientific researchers. Requests must be submitted in writing to the Chief Investigator, including details about the investigator’s background and the intended purpose of the data. Each request will be evaluated based on the proposed analyses, with potential uses likely to include meta-analyses, for example.

The study’s Patient Information Sheets and consent forms explicitly mention the availability of anonymized data, a process approved by the relevant ethics committees.

## Collaborators

KCMC:

Dr Gileard Masenga (Hospital Director); Dr William Makupa (Head of Department); Dr Einoti Matayan (Primary Investigator); Dr Tara Mtuy (Study Co-Ordinator); Mr Saniru Kimweri (Microbiologist); Dr Elisante Muna, Dr Japhet Bright (Investigators); Ms Aiweda Malisa (Research Nurse); Mr Alex Pallangyo (Research Assistant); Dr James Bwire (Opthalmology Resident); Dr Rosena Shao (Opthalmologist); Ms Bibiana Allagwa and Ms Upendo Mmary (Optometrists); Ms Aspreda Mlay and Mr Peter Mushi (Pharmacists); Ms Pulkeria Marandy (Counselor)

MUST:

Dr John Onyango (Head of Department), Dr Abel Ebong (study doctor), Mr. Francis Orishaba (Microbiologist), Dr Angela Birungi (Clinician), Mr. Manzi Gerald (Pharmacist), Mr Johnson Muiriru (Optometrist) Ms. Pauline Boonabaana (Administrator), Mr. Gilbert Arinda (Research nurse), Mr. Martin Bukenya (Research nurse), Mr. Benard Bainomugisha (Research assistant), Ms. Immaculate (Randomisation), Mr. Ronald Wafula (Logistics).

LSHTM:

Jeremy Hoffman (Primary Investigator); Matthew Burton (Chief Investigator); Astrid Leck (Microbiologist); David Macleod, Helen Weiss (Statisticians); Victor Hu (Investigator); Sarah O’Regan (Administrator)
