# Supplementary material for: Randomised controlled trial of topical combination therapy chlorhexidine 0.2% and natamycin 5% versus topical natamycin 5% alone for fungal keratitis in East Africa: study protocol
Source: Wellcome Open Res. 2025 Mar 28;10:165. [Version 1] doi: 10.12688/wellcomeopenres.21390.1 (PMC12149813; doi:10.12688/wellcomeopenres.21390.1)
Supplement: Peer review history

# Reviewer report: Reviewer response for version 1

**DOI:** 10.21956/wellcomeopenres.23655.r123095

**Published:** 2025-06-09

**Authors:** Jeremy Keenan

**Affiliations:** 1 University of California San Francisco, San Francisco, California, USA

This is a protocol for a randomized trial assessing the role of adjunctive chlorhexidine 0.2% for fungal keratitis. Overall, the proposed study is significant given the problem of fungal keratitis in much of the world. The randomized controlled trial design is solid and builds off previous trials which is strength since this study will be more comparable to previous studies. I only have a few minor comments.

 Fig 1: spell out SCEH

 "Notably, chlorhexidine 0.2% exhibited dependable antifungal activity and showed a more favourable response compared to natamycin 2.5% within a 5-day timeframe" --> I wasn't sure at first if this statement was referring to humans. Perhaps add "in two small, randomized trials done in Bangladesh in the 1990s"?

 "a realistic, contextually relevant and sustainable solution" --> is natamycin plus adjunctive chlorhexidine actually realistic and contextually relevant in a place where many patients cannot afford natamycin? It would seem to be more relevant in a place where people could afford natamycin. Ethically it seems appropriate to treat everyone with natamycin, but I wonder how a positive result here will help a person in Africa who cannot afford natamycin. Would you extrapolate that chlorhexidine monotherapy might be effective, despite the results of the Nepal trial?

 Trial summary: consider mentioning it is a parallel-group trial.

 Inclusion criteria: what is the definition of a positive confocal scan for fungus? (Must there be high confidence that this is fungal versus a scan with possible or suspicious filaments but low confidence.) Who will make this determination and when? Will confocal interpreters be experienced? Who will be reading the smears? Do each of the enrolling centers have dedicated microbiology laboratories, or will this capacity need to be introduced?

 Table 2: does "suppuration" refer to discharge? or the cornea? A minor point but I don't usually assess for an APD at the slit lamp.

 Primary outcome analysis - adjusted: this is not listed under the "secondary analyses of the primary outcome" section. What does that make this analysis? Is it simply a sensitivity analysis? It might be good to name it; I do not think you plan to spend any alpha on this analysis? Not needed for protocol but might be worth thinking about how you will interpret the result if the unadjusted analysis is not significant, but the adjusted analysis is significant.

 Presenting VA by Peek: I did not realize that testing would be performed at the home visit. It might be good to mention in the Loss to follow-up section, or maybe in Table 3 (with an asterisk?) the data that would be collected at the home visit. Also, it is not entirely clear when a home visit would be carried out. Would this only be done if they miss their 3-month visit? Is there any chance you could actually get a BSCVA during a home visit? Could a refractionist bring a trial lens kit to the participant's home?

 There are 14 secondary outcomes, with more than 14 p-values. My sense is that most trialists recommend a short list of secondary outcomes due to concerns about multiplicity. (see https://www.fda.gov/files/drugs/published/Multiple-Endpoints-in-Clinical-Trials-Guidance-for-Industry.pdf) You could consider picking the most important as the official secondary outcomes, and then designating the others as exploratory outcomes? You could also leave the 14 secondary outcomes, but it might be a good idea to add some information about how you would determine statistical significance. Do each of them get an alpha of 0.05?

 QOL: in what languages will these surveys be administered? If in local languages do translations already exist? Or how will they be translated?

 Cost-effectiveness: EQ-5D has no vision dimension as far as I know, so I wonder if it will be hard to detect much of a difference. Fine to stick with EQ-5D but just wanted to point out that alternatively you could consider assessing cost per line of vision (e.g. same outcome as clinical trial) or use a vision-related QOL instrument that can be converted into QALYs. Also, in Table 4 it says costs will be estimated from EQ-5D, but there would be no cost information from the EQ-5D, correct?

 Interim analysis section could use another sentence or two. In the sample size section, you mention O’Brien-Fleming; might be worth mentioning again here or briefly describing how you decided you will spend 0.0008 alpha in the interim analysis. Presumably the study could be stopped for efficacy, harm, or futility, but might be good to state this? I assume the DSMB would make a recommendation?

Is the study design appropriate for the research question?

Yes

Is the rationale for, and objectives of, the study clearly described?

Yes

Are sufficient details of the methods provided to allow replication by others?

Yes

Are the datasets clearly presented in a useable and accessible format?

Not applicable

Reviewer Expertise:

ocular infections, epidemiology, clinical trials, screening

I confirm that I have read this submission and believe that I have an appropriate level of expertise to confirm that it is of an acceptable scientific standard.

# Reviewer report: Reviewer response for version 1

**DOI:** 10.21956/wellcomeopenres.23655.r121479

**Published:** 2025-04-01

**Authors:** Shafi Balal

**Affiliations:** 1 University College London, London, England, UK

The article you provided is a detailed study protocol for a randomised controlled trial (RCT) evaluating the effectiveness of combination therapy using topical chlorhexidine 0.2% and natamycin 5% compared to natamycin 5% alone in the treatment of fungal keratitis in East Africa. Overall, the protocol is comprehensive, clearly structured, and addresses an important clinical question.

Is the study design appropriate for the research question?

Yes

Is the rationale for, and objectives of, the study clearly described?

Yes

Are sufficient details of the methods provided to allow replication by others?

Yes

Are the datasets clearly presented in a useable and accessible format?

Yes

Reviewer Expertise:

Corneal researcher (ophthalmology) - NIHR Doctoral Fellow

I confirm that I have read this submission and believe that I have an appropriate level of expertise to confirm that it is of an acceptable scientific standard.
